# Sirtuin 3-mediated pyruvate dehydrogenase activity determines brown adipocytes phenotype under high-salt conditions

**DOI:** 10.1038/s41419-019-1834-4

**Published:** 2019-08-14

**Authors:** Tong Wei, Gaojian Huang, Penghao Liu, Jing Gao, Chenglin Huang, Mengwei Sun, Weili Shen

**Affiliations:** 1 State Key Laboratory of Medical Genomics, Shanghai Key Laboratory of Hypertension, Department of Hypertension Ruijin Hospital, Shanghai Jiaotong University School of Medicine, Shanghai, China; 2 Key Laboratory of State General Administration of Sport, Shanghai Research Institute of Sports Science, 200030 Shanghai, China

**Keywords:** Cell biology, Molecular biology

## Abstract

Previous study indicated that Sirtuin 3 (SIRT3) is a central regulator of adaptive thermogenesis in brown adipose tissue (BAT). Here we investigate the role of SIRT3 in the modulation of cellular phenotype in BAT under high salt intake (HS). HS downregulated SIRT3 level in BAT, accompanied by decreased oxygen consumption rate, and caused a severe loss of BAT characteristics. Mechanically, SIRT3 interacted with pyruvate dehydrogenase E1α (PDHA1) and deacetylated Lys-83 both in vitro and in vivo under HS. In parallel, HS suppressed salt-induced kinase (Sik) 2 phosphorylation. Silencing Sik2 further diminished SIRT3 activity and enhanced acetylation of PDHA1 K83 level. Reconstruction of SIRT3 restored PDH activity and thermogenic markers expression in differentiated brown adipocytes from SIRT3 knockout (KO) mice. In addition, loss of SIRT3 induced selective remodelling of phospholipids and glycerolipids in BAT exposure to HS. These data indicate that SIRT3 is an essential enzymatic switch that controls brown adipose cell phenotype.

## Introduction

Adipose tissue is an important metabolic regulator of energy balance, which has two functionally distinct types of adipocytes. Those in white adipose tissue (WAT) store excess energy and contain a single large lipid vesicle and few mitochondria, whereas those in brown and brite/beige adipose tissue (BAT) burn excess energy and have multilocular lipid droplets and abundant mitochondria that produce heat by uncoupled respiration^1^. It is confirmed that endocrine and nutritional factors can promote the interconversion of WAT and BAT^2^. Excessive nutrition—including high levels of sugar and saturated fat—contributes to triglyceride (TG) and cholesterol accumulation in adipose tissue, resulting in white adipocyte hypertrophy and BAT to WAT conversion^3^. Furthermore, cold exposure and physical exercise facilitate lipid mobilisation, thereby reducing adipocyte hypertrophy and turning WAT into BAT in obese animals and humans^4–6^. Our previous study showed that a high-cholesterol diet can induce brown-to-white adipocyte conversion^7^. These findings demonstrate that carbohydrate and lipid disorders are related to adipose tissue phenotype and function.

Dietary sodium is an important nutritional factor. High salt (HS) intake is the major contributor to high blood pressure and can lead to cardiovascular disease^8,9^; it not only affects renal sodium and water retention, but also lipid metabolism in WAT. However, the role of HS in lipid metabolism and fat deposition is debated^10^. For instance, both human and animal studies have shown that a HS diet increases WAT mass, adipocyte size and leptin production^11,12^. However, others have demonstrated that salt restriction increased WAT mass, whereas a low-sodium diet decreased insulin sensitivity^13^ and increased serum lipid levels in salt-sensitive and -resistant normotensive adults^14^. As for BAT, only one previous study reported that HS intake induced mitochondrial dysfunction by inhibiting mitochondrial GDP-binding capacity and complex I activity^15^. In addition, HS intake reduced mitochondrial metabolic output in macrophages and cardiac tissue^16,17^. Thus, dietary salt loading can cause mitochondrial dysfunction in different organs.

Sirtuin (SIRT)3 belongs to a family of nicotinamide adenine dinucleotide-dependent deacetylases comprising seven isoforms (SIRT1–7) that localise to the mitochondrial matrix and regulate metabolism, apoptosis and DNA repair^18^. Murine SIRT3 is expressed at high levels in brown and low levels in white fat, respectively. Cold exposure and caloric restriction induce SIRT3 expression^19^, whereas a high-fat diet was shown to suppress the expression of SIRT3, peroxisome proliferator-activated receptor-γ co-activator (PGC)-1α and uncoupling protein (UCP)-1 in brown adipocytes^20^. Thus, SIRT3 activates mitochondrial function and plays an important role in adaptive thermogenesis in BAT. However, whether SIRT3 deficiency leads to mitochondrial dysfunction in brown adipocytes under HS is elusive.

Salt-inducible kinases (Siks) belong to the AMP-activated protein kinase (AMPK) family that regulates whole body energy homoeostasis. Sik1 is a salt-inducible gene that is highly expressed in the adrenal gland^21^. Sik2 is an adipose-specific kinase, whereas Sik3 is ubiquitously expressed in somatic tissues^22^. Sik2 activity was shown to be upregulated by nutrient deprivation, and Sik2 overexpression inhibited the expression of lipogenic genes and reduced TG content in adipocytes^23^. In addition, Sik2 is downregulated in adipose tissue from obese individuals^24^. The link between the biological function of Sik2 and SIRT3 under HS stress is intriguing.

In this study, we found that SIRT3 decreased in BAT of mice subjected to high salt intake. The expression of SIRT3 correlated with the expressions of genes related to mitochondrial function and lipid droplet size. Then we determined the role of SIRT3 in the regulation of BAT conversion to WAT in SIRT3 knockout (KO) mice on a HS diet.

## Material and methods

### Materials

Collagenase type II, insulin, isobutylmethylxanthine, dexamethasone, 3,3′,5-triiodo-l-thyronine (T3), rosiglitazone and UCP-1 antibody were purchased from Sigma-Aldrich (St. Louis, MO, USA). NaCl was from Xilong Chemicals (Guangzhou, China). Sodium dichloroacetate (DCA) was from J&K Scientific (Beijing, China). Antibody against complex I was from Life Technologies (Carlsbad, CA, USA). Antibodies against TOM-20 and PGC-1α were from Santa Cruz Biotechnology (Dallas, TX, USA). Antibodies against SIRT3, Sik2, perilipin-1, PDHA1 and acetyl-lysine were from Cell Signaling Technology (Beverly, MA, USA). Phosphorylated-Sik2 was purchased from Invitrogen (Carlsbad, CA, USA). The acetylated PDHA1 K83 antibody and peptide were synthesised by Huaan Biotechnology (Hangzhou, China). Secondary antibodies including peroxidase-conjugated rabbit anti-goat IgG, goat anti-rabbit IgG and rabbit anti-mouse IgG were from Life Technologies. ShRNA targeting Sik2 (Sik2-shRNA) and SIRT3-overexpressing lentivirus were synthesised by Genechem (Shanghai, China). Alexa Fluor 555-labelled anti-mouse IgG, Alexa Fluor 488-labelled anti-rabbit IgG, Alexa Fluor 594-labelled anti-rabbit IgG and MitoTracker Red were from Life Technologies. Primers were purchased from SBS Genetech (Shanghai, China). Boron-dipyrromethene (BODIPY) was from Molecular Probes (Eugene, OR, USA). Cell culture reagents were from Hyclone (Logan, UT, USA).

### Animal models

SIRT3-KO mice on a 129/SV background (stock no. 027975) were purchased from the Jackson Laboratory (Bar Harbor, ME, USA) and backcrossed onto the C57BL/6 background for ten generations; WT C57BL/6 mice served as a control. Male 8-week-old SIRT3-KO and age-matched WT mice were given either NS or HS (1% NaCl) tap water. The mice were maintained in a temperature- and humidity-controlled animal facility on a 12:12-h light/dark cycle, with free access to water and standard rodent chow. After 6 weeks, mice were anesthetised by intraperitoneal injection of ketamine/xylazine (100 mg/10 mg/kg) and interscapular BAT depots were obtained and stored at −80 °C until analysis. All procedures were approved by the Institutional Animal Care and Use Committee at Shanghai Jiaotong University and were performed in accordance with the Guide for the Care and Use of Laboratory Animals (NIH Publication, 8th edition, 2011).

### Physiological measurements

Systolic blood pressure was measured weekly with a non-invasive tail-cuff system (BP-2000; Visitech Systems, Apex, NC, USA). Water intake was recorded daily and body weight was measured every other day for each mouse. Sodium concentration in serum was assayed with commercially available analysis kits according to the manufacturer’s instructions (Jiancheng Biochemical Inc., Nanjing, China).

### Electron microscopy

Fresh adipose tissue samples were fixed in 2.5% (v/v) glutaraldehyde in phosphate-buffered saline, postfixed in 4% (w/v) osmium tetroxide and embedded in Epon resin. Ultrathin sections (50–80 nm thick) were cut and stained with lead citrate and uranyl acetate and observed with a transmission electron microscope (CM 10; Philips, Eindhoven, the Netherlands).

### Isolation of SVF and induction of brown adipocyte differentiation

The stromal vascular fraction (SVF) from excised interscapular, subscapular and axillary BAT depots was prepared and differentiation was induced with insulin (5 μg/ml), isobutylmethylxanthine (0.5 mmol/l), dexamethasone (1.0 μmol/l), T3 (10 nmol/l) and rosiglitazone (1.0 μmol/l) in Dulbecco’s Modified Eagle’s Medium (DMEM) supplemented with 10% fetal bovine serum (FBS) for 48 h. The culture medium was replaced with DMEM supplemented with 10% FBS and insulin, T3 and rosiglitazone for another 6 days^7^. The cells were infected with lentivirus expressing murine SIK2-shRNA, the SIRT3 coding sequence, or green fluorescent protein-expressing control vector. Transduced and differentiated brown adipocytes were stimulated with NaCl (40 mmol/l) for 24 h or left unstimulated.

### Histological and immunofluorescence analyses

Interscapular BAT specimens were fixed in 4% paraformaldehyde solution and embedded in paraffin or Tissue-Plus O.C.T. compound (Scigen Scientific, Gardena, CA, USA). Frozen 5-μm serial sections were cut and stained with hematoxylin and eosin or used for immunofluorescence analysis. Sections were incubated with antibodies against SIRT3 (1:300), TOM-20 (1:200), UCP-1 (1:300) and perilipin-1 (1:400) followed by Alexa Fluor 594-conjugated anti-rabbit or Alexa Fluor 488-conjugated anti-rabbit or anti-mouse IgG. In addition, 10-μm frozen tissue sections were stained with BODIPY to detect lipid droplets. For cells staining, differentiated brown adipocytes were fixed with mixture of methanol and acetone and blocked with 0.01% Triton-BSA. Cells were incubated with complex I (1:300), UCP-1 (1:300) and PDHA1 K83-Ac (1:300) antibodies at 4 °C overnight followed by secondary antibodies. After nuclei staining, coverslips were observed under fluorescence microscope (Carl Zeiss, Milan, Italy).

### Western blot analysis

Tissue and cell lysates (10 μg of protein per lane) were separated by 10% sodium dodecyl sulfate polyacrylamide gel electrophoresis and then transferred to a polyvinylidene difluoride membrane, which was blocked with 5% (w/v) non-fat dried skimmed milk powder in Tris-buffered saline with 0.01% Tween 20 (TBST) for 1 h and then incubated overnight at 4 °C with primary antibodies against SIRT3 (1:1000), UCP-1 (1:2000), TOM-20 (1:1000), PGC-1α (1:1000), complex I (1:4000) and actin (1:8000). The membrane was washed with TBST and then incubated with appropriate peroxidase-conjugated secondary antibody for 1 h. After washing, the blot was developed by electro-chemiluminescence and protein band intensity was quantified by scanning densitometry (Model 670; Bio-Rad, Hercules, CA, USA).

### RNA isolation and real-time PCR

Total RNA was extracted using TRIzol reagent (Life Technologies) according to the manufacturer’s protocol, and 1.0 μg was reverse-transcribed into cDNA. Quantitative real-time PCR was performed on a Step One Plus Real-Time PCR system (Applied Biosystems, Foster City, CA, USA) with SYBR Green Real-time PCR Master Mix Plus (Toyobo, Osaka, Japan). Relative differences in transcript levels were calculated by the 2^−ΔΔCT^ method, with the mouse 18S rRNA gene serving as the endogenous reference. The primers used are shown in Table 1.

**Table 1:** Primer sequences used for PCR were as follows

	Forward	Reverse
Nrf1	5′-GCCGTCGGAGCACTTACT-3′	5′-CTGTTCCAATGTCACCACC-3′
Tfam	5′-CGCAGCACCTTTGGAGAA-3′	5′-CCCGACCTGTGGAATACTT-3′
D-loop	5′-AATCTACCATCCTCCGTG-3′	5′-GACTAATGATTCTTCACCGT-3′
18S rRNA	5′-CATTCGAACGTCTGCCCTATC-3′	5′-CCTGCTGCCTTCCTTGGA-3′

### Total DNA isolation and PCR

Total DNA was extracted using a QIAamp DNA Mini kit (Qiagen, Valencia, CA, USA), and quantitative PCR was performed as previously described^25^ using primers amplifying mitochondrial D-loop and 18S rDNA. The ratio of mitochondrial D-loop to 18S rDNA was calculated.

### PDH activity assay

The activity of PDH was analysed using the PDH activity assay kit (Sigma), and the assay was performed according to the manufacturer’s protocol. Cells (1 × 10^6^) were homogenised in 100 μl of ice-cold PDH assay buffer and centrifuged at 10,000 *g* for 5 min to remove insoluble material. Pyruvate dehydrogenase activity is determined using a coupled enzyme reaction, which results in a colorimetric (450 nm) product proportional to the enzymatic activity present. The experiments were performed in triplicate and the results were compared with the control values.

### Oxygen consumption rate analysis

The oxygen consumption rate was measured using a Seahorse XFe96 Extracellular Flux Analyzer (Seahorse Bioscience, North Billerica, MA). Isolated SVF cells were seeded in cell culture plates followed by 10 days differentiation. The cells were infected with SIK2-shRNA, LV-SIRT3, or control vector at 4th day. After NaCl stimulation, the medium was replaced with XF assay medium, containing 25 mmol/l glucose, 1 mmol/l sodium pyruvate, and 2 mmol/l glutamine, and incubated in a CO_2_ free 37 °C incubator for 1 h. The oxygen consumption rate was analysed by sequentially adding oligomycin (1 μmol/l), FCCP (2 μmol/l), and rotenone/antimycin A(1 μmol/l), and the basal respiration, ATP-linked OCR, maximal respiration and reserved capacity were calculated from the primary data.

### LC–MS/MS analysis

Murine BAT separated from WT and SIRT3-KO mice were provided for proteomic quantification of lysine acetylation. Using TMT labelling and affinity enrichment followed by high-resolution LC–MS/MS analysis, quantitative lysine acetylome analysis was performed. Altogether, 1331 lysine acetylation sites in 431 protein groups were identified from mouse, among which 916 sites in 305 proteins were quantified. The mass spectrometry of modified PDHA1 sequence was showed.

### Extraction of lipids from BAT and lipidomics analysis

Frozen BAT (100 mg) was homogenised in chloroform:methanol solution (1:2, v/v)^26^ and the homogenate was centrifuged at 3000 × *g* for 5 min at 4 °C to separate the organic and aqueous layers. The former was removed and concentrated under N_2_ gas and then dissolved in 250 μl of chloroform:methanol solution (2:1, v/v) before analysis by LC–MS. Chromatographic separation was performed with an LC-30A system (Shimadzu, Kyoto, Japan) using an Acquity ultra-high performance LC HSS T3 (150 × 2.1 mm, 1.8 µm; Waters, Milford, MA, USA) column maintained at 40 °C. A 5-μl volume of each sample was injected via the auto-sampler, with the temperature set at 4 °C. Acetonitrile/methanol/water at a 1:1:1 (v/v/v, with 5 mmol/l ammonium acetate) ratio (A) and isopropanol/acetonitrile at a 1:1 (v/v) ratio (B) were used for gradient elution of analytes at a flow rate of 0.3 ml/min. An increasing linear gradient of solvent B (v/v) was used as follows: 0–0.5 min, 20% B; 0.5–1.5 min, 20–40% B; 1.5–3 min, 40–60% B; 3–13 min, 60–98% B; 13–13.1 min, 98–20% B; 14–15 min, 20%. Electrospray ionisation tandem MS was performed on an AB 5600+ mass spectrometer (AB SCIEX, Framingham, MA, USA) under a spray voltage of 5.50 kV in positive mode and −4.50 kV in negative mode. Gases 1 and 2 were both set at 50 psi, and curtain gas was 35 psi. The source temperature was 500 °C. The mass analyser scanned over a mass range of m/z 100–1500 for a full scan at collision energy of 45 eV. Dynamic exclusion was implemented.

### Statistical analysis

Data are presented as the mean ± SEM using GraphPad Prism 6.0. Statistical significance was evaluated using one-way analysis of variance (ANOVA) followed by post hoc comparisons with Tukey’s HSD test. Differences between two groups were analyzed using Student’s *t*-test, *p* < 0.05 was considered statistically significant.

## Results

### SIRT3 level is reduced in BAT after 6 weeks of HS intake

**Fig. 1:**
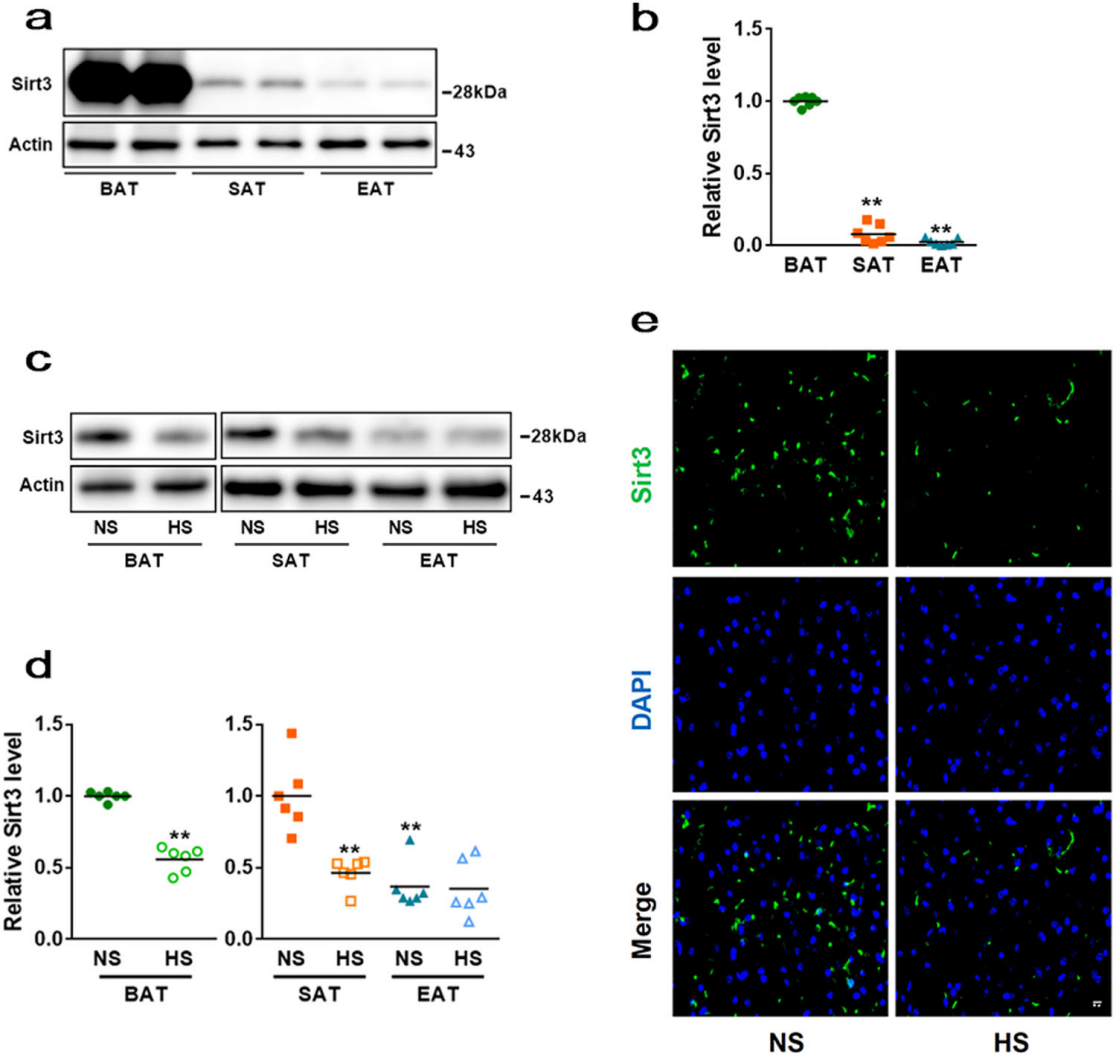
HS intake inhibits SIRT3 expression in BAT. Adipose tissue was harvested from two WAT depots and interscapular BAT of C57BL/6 mice. **a** Western blot analysis of SIRT3 expression in BAT and WAT. **b** Semi-quantitative analysis of SIRT3 level in BAT and WAT. **c** Western blot images of SIRT3 level in BAT and WAT of C57BL/6 mice with or without HS intake. **d** SIRT3 level was normalised to that of actin and is presented as fold increase relative to BAT NS and SAT NS group, respectively. **e** Tissue sections were harvested from BAT of the C57BL/6 mice and labelled with antibody against SIRT3 (green); nuclei were stained with 4′,6-diamidino-2-phenylindole (DAPI; blue). (*n* = 3/group). Scale bar: 50 μm. Data are presented as mean ± SEM (*n* = 10/group). **p < 0.01 vs. relative control

To examine the effects of HS on SIRT3 expression, C57BL/6 mice were subjected to HS (1% NaCl in tap water) intake for 6 weeks. We then isolated adipose tissue from two WAT depots and the interscapular BAT of C57BL/6 mice, and analysed SIRT3 expression by western blotting. The results showed that SIRT3 is abundantly expressed in BAT compared with subcutaneous (SAT) and epididymis (EAT) WAT (Fig. 1a, b). After 6 weeks of HS intake, SIRT3 protein level was markedly reduced in BAT and SAT; whereas no obvious difference was observed in EAT (Fig. 1c, d). Immunofluorescence analysis also confirmed that SIRT3 protein level in BAT was decreased after HS administration (Fig. 1e). These results indicate that HS causes a decline in SIRT3 level in BAT.

### SIRT3 deficiency promotes HS-induced BAT lipid droplet remodelling

**Fig. 2:**
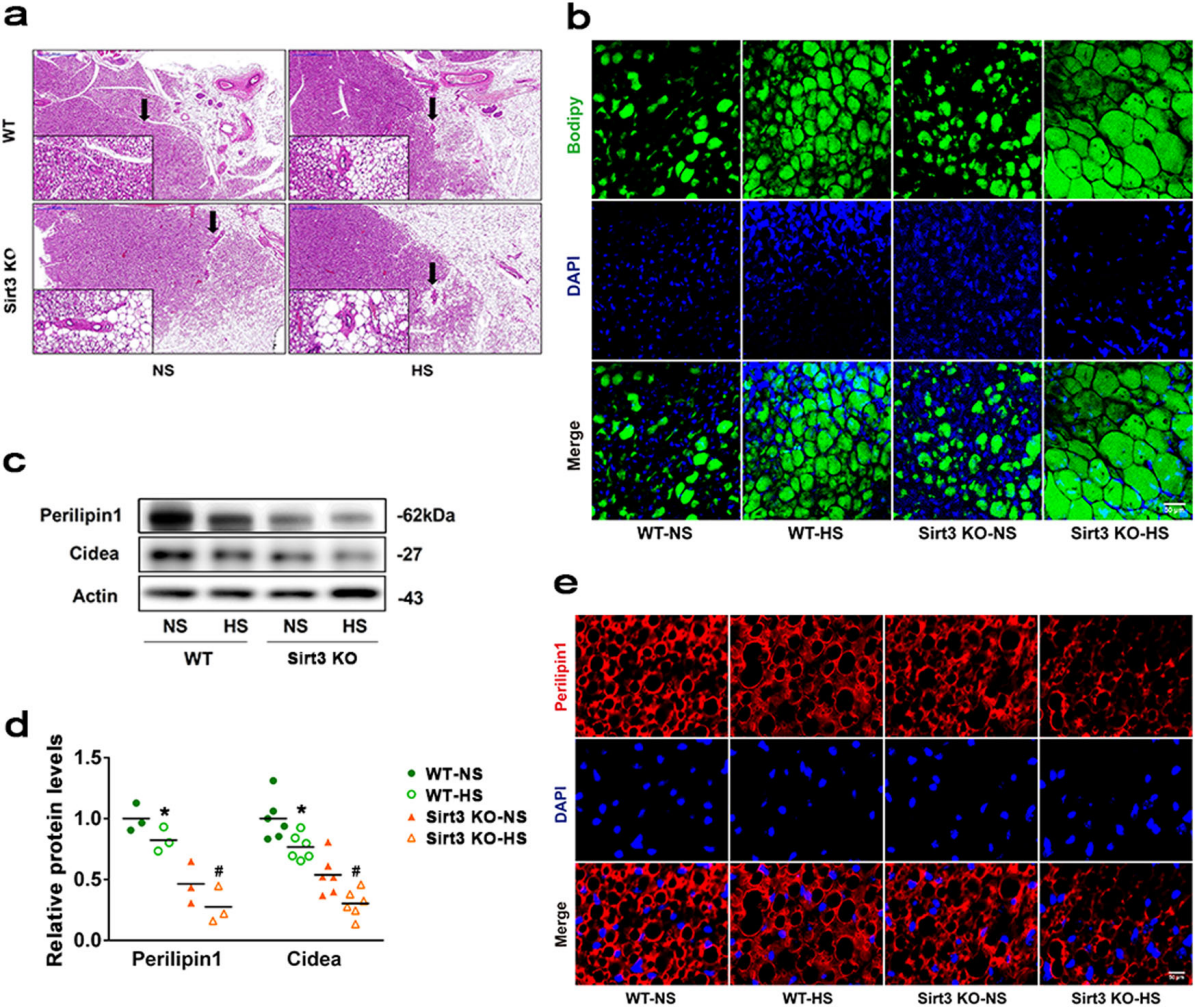
SIRT3 deficiency promotes HS-induced BAT lipid droplet remodelling. **a** BAT sections were harvested from WT and SIRT3-KO mice and stained with hematoxylin and eosin (*n* = 3/group). Scale bar: 500 μm. **b** BAT sections were harvested from WT and SIRT3-KO mice and stained with BODIPY (green) and 4′,6-diamidino-2-phenylindole (DAPI; blue) (*n* = 3/group). Scale bar: 50 μm. **c** Representative western blot **d** and quantitative analysis of perilipin-1 and Cidea levels in BAT, shown as fold increase relative to the WT NS group. Data are presented as mean ± SEM (*n* = 10/group); *p < 0.05 vs. genotype-matched NS mice; ^#^*p* < 0.05 vs. WT HS mice. **e** BAT sections were harvested from WT and SIRT3-KO mice and labelled with an antibody against perilipin-1 (red); nuclei were stained with DAPI (blue). (*n* = 3/group). Scale bar: 50 μm

To investigate the role of SIRT3 in regulating HS-induced BAT remodelling, SIRT3 KO and wild-type (WT) mice were challenged with HS intake for 6 weeks, an increase in water consumption was observed in both groups, but was especially pronounced in SIRT3-KO mice. HS treatment induced a significant increase in body weight gain in WT and SIRT3-KO mice compared with their normal salt (NS)-treated controls, respectively. No difference in blood pressure was observed between WT and SIRT3-KO mice under NS or HS. Serum sodium level was higher in HS group than in NS group, but without significant difference (Supplementary Fig 1). BAT in WT HS mice had a WAT-like appearance including larger, unilocular adipocytes as compared with those in WT NS mice. Similarly, SIRT3-KO mice showed the whitening of BAT (Fig. 2a), which was accompanied by a switch from multilocular to unilocular morphology and increased lipid content (Fig. 2b). HS intake induced BAT whitening in SIRT3-KO mice, with a concomitant decline in the levels of perilipin-1 and Cidea, which are localised on the surface of lipid droplets (Fig. 2c–e). These results indicate that SIRT3 deficiency promotes HS-induced BAT lipid droplet remodelling.

### SIRT3 deficiency exacerbates impairment of BAT mitochondrial biogenesis

**Fig. 3:**
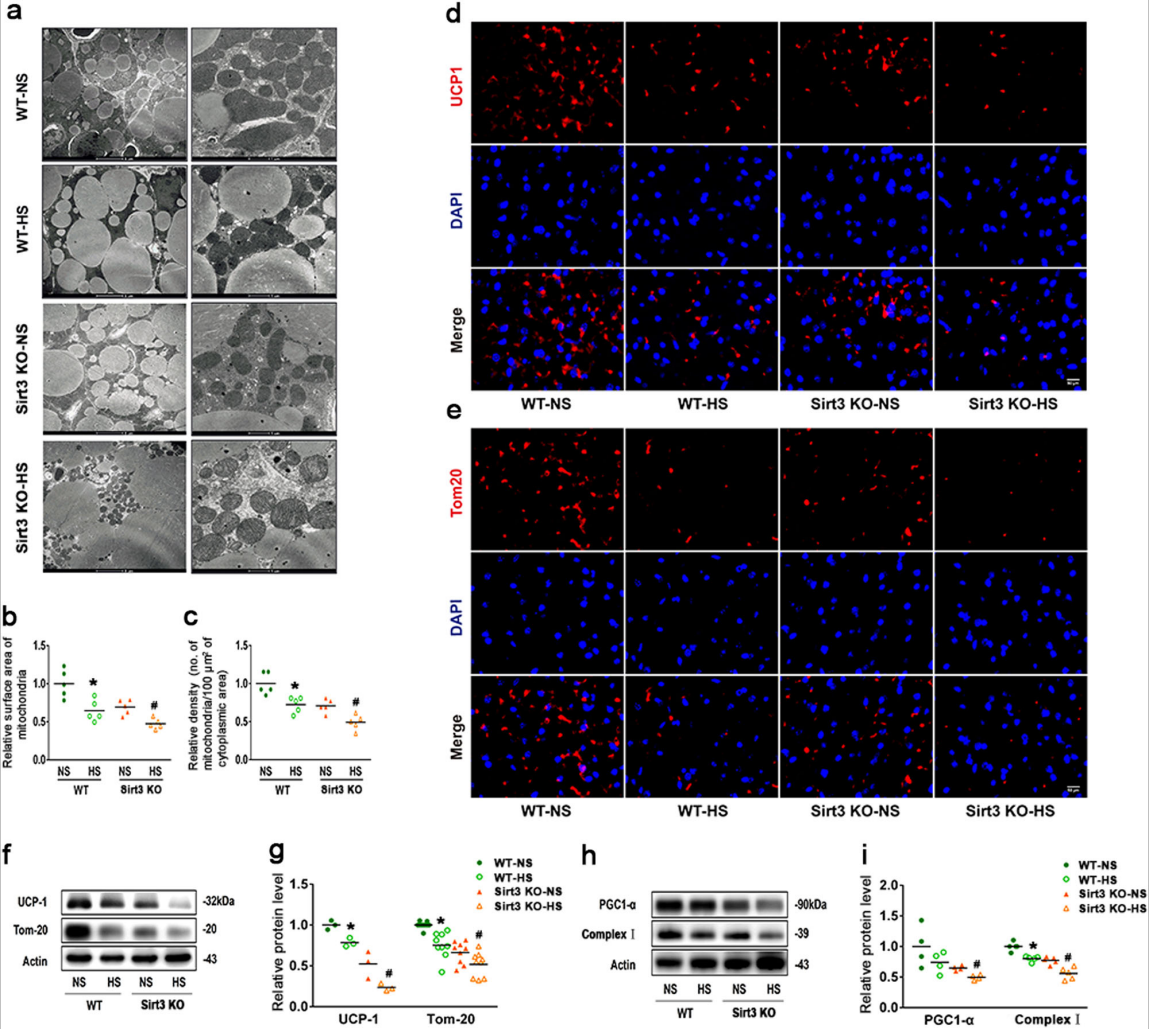
SIRT3 deficiency enhances the suppression of BAT mitochondrial biogenesis. **a** Electron micrograph of mitochondria from BAT of WT and SIRT3-KO mice (magnification, ×10,000 and ×20,000). **b**, **c** Morphometric analysis of mitochondrial surface area and density. **d** BAT sections were prepared from WT and SIRT3-KO mice and labelled with an antibody against UCP-1 (red); nuclei were stained with 4′,6-diamidino-2-phenylindole (DAPI; blue) (*n* = 3/group). Scale bar: 50 μm. **e** BAT sections were labelled with an antibody against TOM-20 (red); nuclei were stained with DAPI (blue) (*n* = 3/group). Scale bar: 50 μm. **f** Representative western blot and **g** quantitative analyses of UCP-1 and TOM-20. **h** Representative western blot and **i** quantitative analyses of PGC-1α and complex I levels, shown as fold increase relative to the WT NS group. Data represent as mean ± SEM (*n* = 10/group). *p < 0.05 vs. genotype-matched NS mice; ^#^*p* < 0.05 vs. WT HS mice

BAT is characterised by abundant mitochondria that produce heat via uncoupled respiration. To confirm the effects of HS intake on mitochondrial biogenesis in BAT, we first examined changes in mitochondrial mass and density in WT and SIRT3-KO mice with or without HS intake. An electron microscopy analysis revealed that BAT in WT NS mice was composed of multilocular lipid droplets with abundant mitochondria that were spherical, large, and packed with laminar cristae (Fig. 3a). Mitochondria in SIRT3-KO mice had abnormal morphology and were larger with less densely packed cristae. A quantitative analysis revealed that HS intake decreased total mitochondrial area and number in the BAT of WT mice. HS intake caused almost complete swelling of mitochondria in the BAT of SIRT3-KO mice; the mitochondria also exhibited a low electron density matrix and reduced mass (Fig. 3a–c). Immunofluorescence and western blot analyses confirmed the downregulation of the BAT proteins UCP-1 and translocase of outer mitochondrial membrane (TOM)-20 (Fig. 3d–g).

PGC-1α regulates mitochondrial biogenesis to control cellular bioenergetics. Protein levels of PGC-1α and complex-1 were downregulated in BAT of SIRT3-KO NS mice as compared with WT NS mice. The HS-induced reduction of mitochondrial mass was associated with downregulation of PGC-1α protein, which was greater in SIRT3-KO mice than in controls (Fig. 3h, i). These results indicate that SIRT3 deficiency exacerbates the dysregulation of BAT mitochondrial biogenesis.

### SIRT3 deficiency promotes the conversion of BAT to WAT under HS conditions

**Fig. 4:**
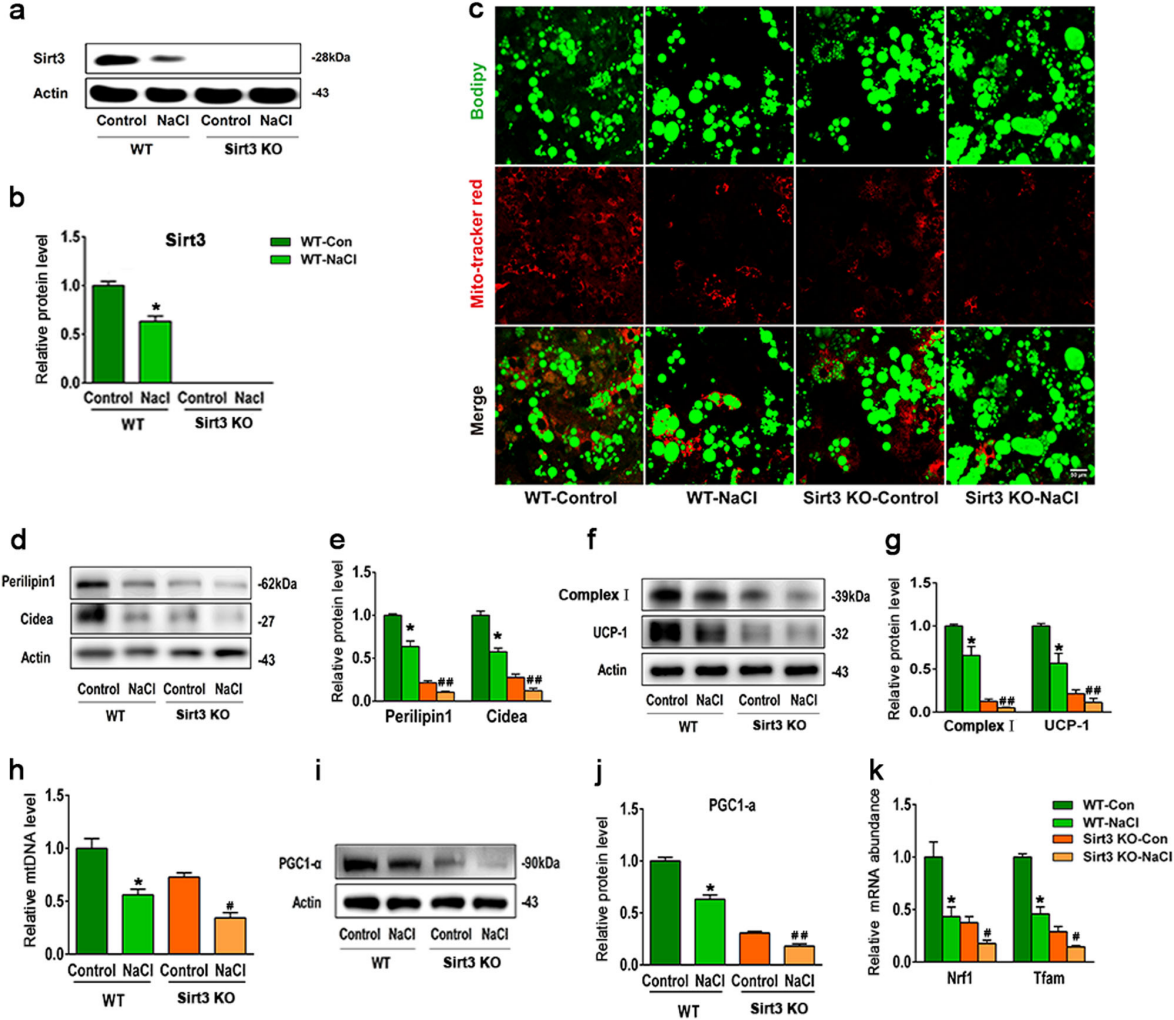
SIRT3 deficiency accelerates the conversion of BAT to WAT. **a** Representative western blot and **b** semi-quantitative analysis of SIRT3 level in brown adipocytes from WT and SIRT3-KO mice. **c** Brown adipocytes from WT and SIRT3-KO mice were stained with BODIPY (green) and MitoTracker Red (red). Scale bar: 50 μm. **d** Representative western blot and **e** semi-quantitative analysis of perilipin-1 and Cidea levels in brown adipocytes. **f** Representative western blot and **g** semi-quantitative analysis of UCP-1 and complex I levels in brown adipocytes. **h** D-loop to 18S rDNA ratio. **i** Representative western blot and **j** semi-quantitative analysis of PGC-1α level. **k** mRNA abundance of Nrf1 and TFAM. Results are shown as fold increase relative to the WT control and represent mean ± SEM of four independent experiments. *p < 0.05 vs. WT control; ^#^*p* < 0.05, ^##^*p* < 0.05 vs. WT NaCl

To explore the relationship between HS intake and the phenotypic transition of brown adipocytes, primary SVF cells from BAT depots were induced to differentiate into brown adipocytes and then incubated with complete medium in the presence/absence of 40 mmol/l additional NaCl. As expected, exposure to HS reduced SIRT3 protein level in BAT from WT mice (Fig. 4a, b). We carried out immunofluorescence analysis with BODIPY and MitoTracker Red to determine the relative amount and size of lipid droplets and to quantify mitochondria in adipocytes, respectively. Brown adipocytes from WT mice had fewer mitochondrion and larger lipid droplets after incubation with HS relative to control cells, an effect that was exacerbated in brown adipocytes from SIRT3-KO mice (Fig. 4c). Consistent with these morphological alterations, HS decreased the levels of lipid droplet-associated (perilipin-1 and Cidea) and mitochondrial (UCP-1 and complex-1) protein markers along with mitochondrial DNA copy number in brown adipocytes from WT mice; these changes were more pronounced in SIRT3-KO mice (Fig. 4c–h). Brown adipocytes of WT mice showed decreased levels of PGC-1a, nuclear respiratory factor (Nrf)1 and transcription factor A mitochondrial (Tfam) following exposure to HS, especially in BAT of SIRT3-KO mice (Fig. 4i–k). These results indicate that SIRT3 deletion promotes HS-induced brown-to-white adipocyte conversion by inhibiting mitochondrial biogenesis and perilipin-1 expression.

### Restoring SIRT3 prevents HS-induced BAT to WAT conversion through improving mitochondrial respiration

**Fig. 5:**
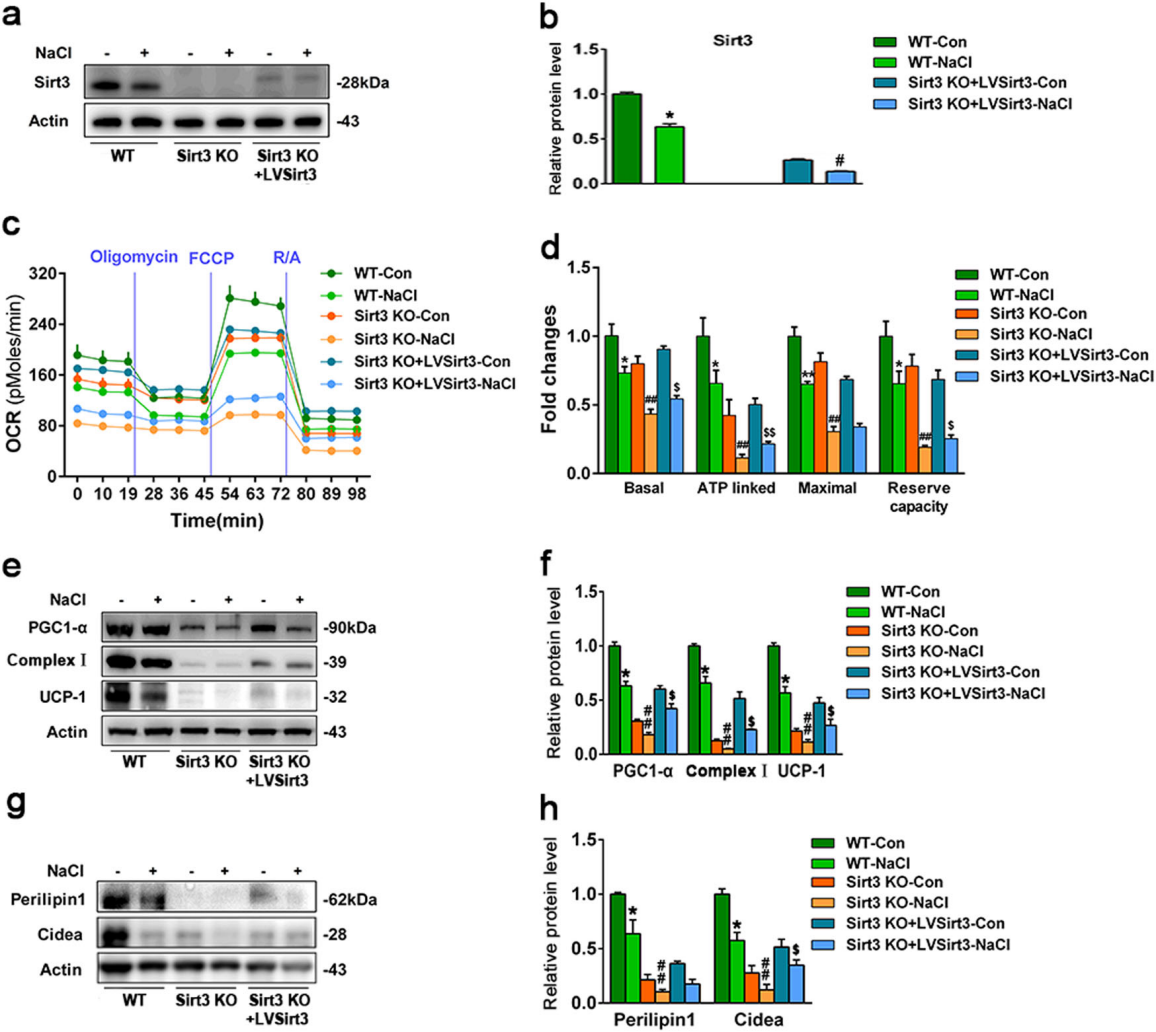
Restoring SIRT3 expression prevents high salt-induced BAT conversion to WAT. **a** Representative western blot and **b** quantitative analysis of SIRT3 in WT, SIRT3-KO and SIRT3-reconstruction brown adipocytes treated with or without NaCl. **c** The oxygen consumption rate (OCR) of brown adipocytes measured with XFe96 Extracellular Flux Analyzer. **d** Statistical analysis of OCR. **e** Representative western blots and **f** semi-quantitative analyses of PGC-1α, UCP-1 and complex I; and **g**, **h** perilipin-1 and Cidea levels in WT, SIRT3-KO and SIRT3-reconstruction brown adipocytes treated with or without NaCl. Results are shown as fold increase relative to the WT control and are presented as mean ± SEM of four independent experiments. *P < 0.05, **P < 0.01 vs. WT control; ^#^*P* < 0.05, ^##^*P* < 0.01 vs. WT-NaCl. ^$^*P* < 0.05, ^$$^*P* < 0.01 vs. SIRT3-KO-NaCl

To investigate the mechanism of action of SIRT3 in mitochondrial biogenesis and modulation of lipid droplet size, we determined overall mitochondrial function using a Seahorse XFe96 Extracellular Flux Analyzer to measure the oxygen consumption rate. SIRT3 was reintroduced into the SIRT3-KO cells to a level comparable with the endogenous SIRT3 (Fig. 5a, b), which restored mitochondrial respiration, as the basal oxygen consumption, ATP-linked OCR, maximal respiration and reserved capacity all increased compared with SIRT3-KO cells (Fig. 5c, d). In parallel, the expression of the mitochondrial proteins (UCP-1, complex I) and lipid droplet-binding proteins (perilipin-1, Cidea) were elevated in SIRT3 preserved cells (Fig. 5e–h), suggesting that SIRT3 may stimulate mitochondrial biogenesis and suppress the conversion of BAT to WAT under HS conditions through promoting mitochondrial respiration.

### SIRT3 interacted with and deacetylated PDHA1 lysine 83

**Fig. 6:**
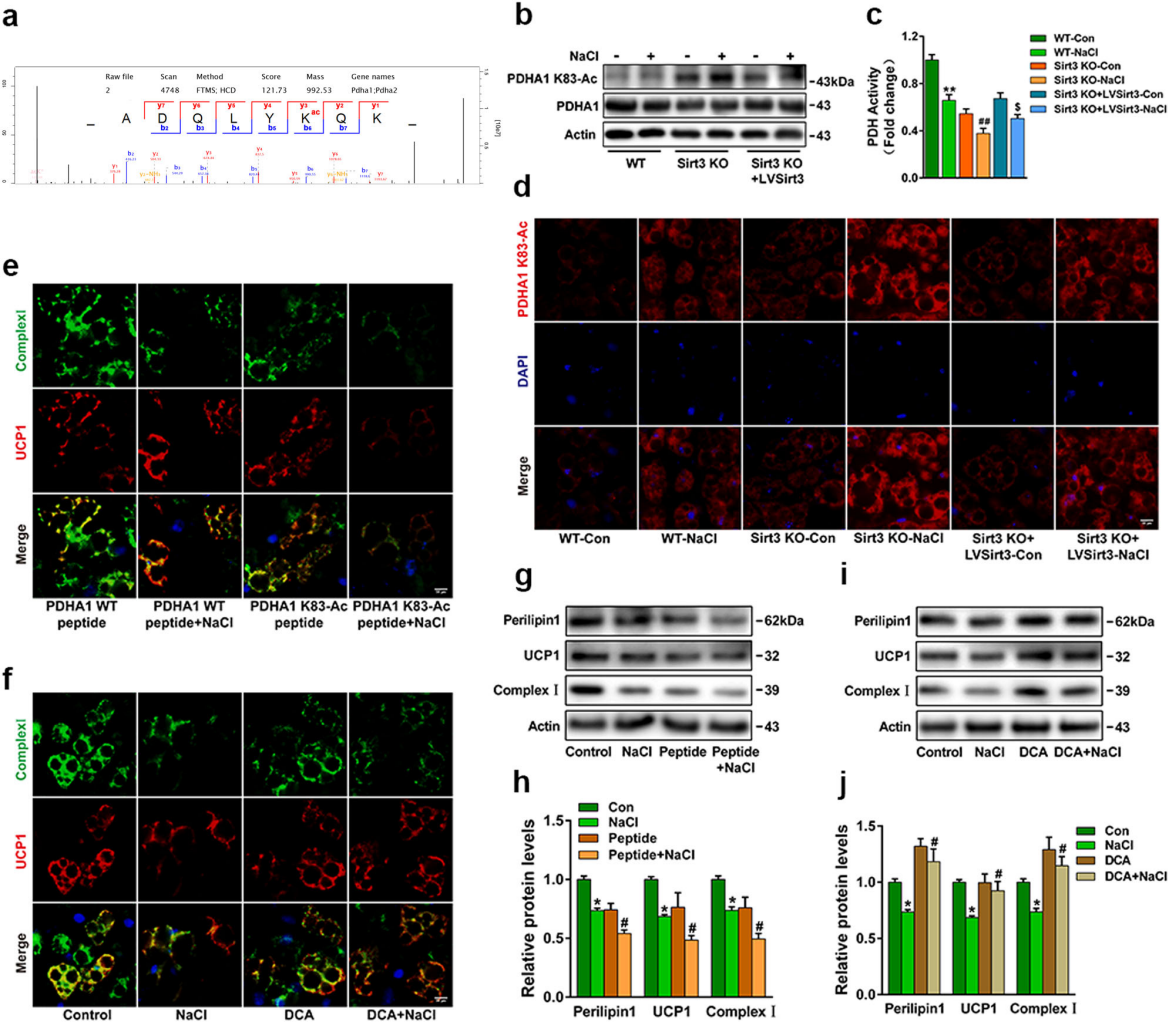
Acetylation of PDHA1 K83 alters mitochondrial function of brown adipocytes. **a** The mass spectrum of PDHA1 acetylation sites in SIRT3-KO mice compared with WT mice. **b** The expression of PDHA1 K83-Ac and **c** PDH activity in WT, SIRT3-KO and SIRT3-reconstruction brown adipocytes treated with or without NaCl. **d** Differentiated brown adipocytes stained with PDHA1 K83-Ac antibody (red); nuclei were stained with DAPI (blue). Scale bar: 50 μm. Representative images of brown adipocytes pretreated with PDHA1 K83-Ac peptide **e** or DCA **f** labelled with complex I (green) and UCP-1 (red). Scale bar: 50 μm. **g** Representative western blots and **h** semi-quantitative analyses of perilipin-1, UCP-1 and complex I in PDHA1 K83-Ac peptide or **i**, **j** DCA pretreated brown adipocytes. Results are shown as fold increase relative to the WT control and are presented as mean ± SEM of four independent experiments. *P < 0.05, **P < 0.01 vs. WT control; ^#^*P* < 0.05, ^##^*P* < 0.01 vs. WT-NaCl. ^$^*P* < 0.05 vs. SIRT3-KO-NaCl

To further determine the mechanism of SIRT3 in regulating mitochondrial respiration, we used acetyl-lysine antibody affinity purification and mass spectrometry to identify the difference of acetylated proteins between WT and SIRT3-KO cells. Pyruvate dehydrogenase E1 alpha 1(PDHA1) plays a vital role in regulating cellular respiration, a gatekeeper between glycolysis pathway and citric acid cycle. Mass spectrometry analysis identified that K83 of PDHA1 can be acetylated (peptide ADQLYKQK) (Fig. 6a). We next generated an antibody specifically recognising K83-acetylated PDHA1 by immunising rabbits with K83-acetylated peptide (CLKADQLYK(Ac)QKIIRG). Using the acetyl-specific antibody, we found that K83 acetylation of PDHA1 increased in NaCl-treated WT-cells. Ablated SIRT3 further elevated PDHA1 K83-Ac and inhibited PDH activity. Conversely, reintroduced SIRT3 decreased PDHA1 K83-Ac and restored PDH activity (Fig. 6b–d). To examine the effects of PDHA1 K83 acetylation on brown adipocytes, we treated cells with specific acetyl-K83 PDHA1 peptide. Western blot and immune fluorescence analysis exhibited that K83 acetylation aggravated NaCl-induced loss of UCP-1 and complex I (Fig. 6e, g, h). DCA, an inhibitor of pyruvate dehydrogenase kinase (PDK), reversed NaCl-induced brown adipocytes phenotype conversion by promoting perilipin-1, UCP-1 and complex I in DCA-treated cells (Fig. 6f, i, j), indicating that PDH activation could maintain the function of brown adipocytes.

### Sik2 signalling is required for SIRT3-induced mitochondrial biogenesis

**Fig. 7:**
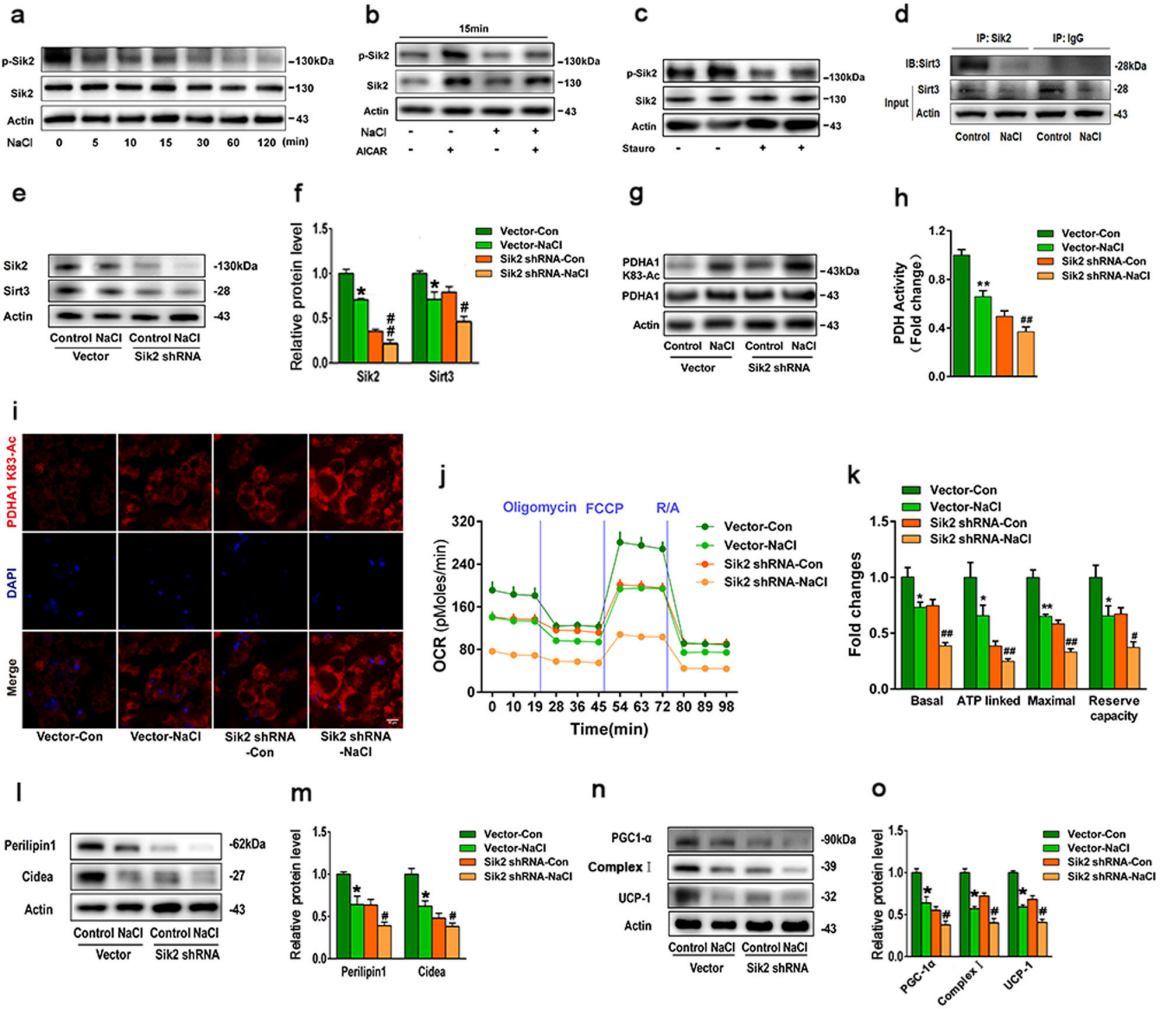
Sik2 signalling is required for SIRT3-induced mitochondrial biogenesis. **a** Representative western blot images of p-Sik2 and Sik2 in time response. **b** Representative western blot images of p-Sik2 and Sik2 in brown adipocytes treated with or without AICAR. **c** Representative bands of p-Sik2 and Sik2 treated with staurosporine. **d** Representative western blot of SIRT3 level following immunoprecipitation with anti-SIK2 antibody in brown adipocytes from WT mice. **e** Representative western blots and **f** semi-quantitative analyses of Sik2 and SIRT3 in brown adipocytes from WT mice infected with Sik2-shRNA or scrambled shRNA vector. **g** Representative western blot of PDHA1 K83-Ac and **h** PDH activity in Sik2-shRNA or vector-shRNA infected brown adipocytes. **i** Immunofluorescence images of SIK2-shRNA infected brown adipocytes stained with PDHA1 K83-Ac antibody (red); nuclei were stained with DAPI (blue). Scale bar: 50 μm. **j** The oxygen consumption rate (OCR) and **k** statistical analysis of Sik2-shRNA infected brown adipocytes. **l**, **m** Representative western blot and semi-quantitative analyses of perilipin-1 and Cidea; and **n**, **o** PGC-1α, UCP-1 and complex I levels in brown adipocytes from WT mice infected with Sik2-shRNA or scrambled shRNA vector. Results are shown as fold increase relative to vector control, and are presented as mean ± SEM of four independent experiments. *P < 0.05, **P < 0.01 vs. vector control; ^#^*P* < 0.05, ^##^*P* < 0.01 vs. vector NaCl

We observed that Sik1 was abundantly expressed in adrenal glands and was upregulated in response to HS intake, whereas Sik1 was almost undetectable in BAT under NS or HS conditions. Sik2 was highly expressed in BAT and was decreased in response to HS intake for 6 weeks, while Sik2 expression was low in the adrenal glands irrespective of salt concentration (Supplementary Fig 2). As shown in Fig. 7a–c, NaCl inhibited Sik2 phosphorylation at Thr175/Thr163 in time-dependent manner. Then we demonstrated that the AMPK activator 5-amino-4-imidazolecarboxamide (AICAR) could induce p-Sik2 (Thr175/Thr163), while pretreatment with NaCl resulted in reduction of p-Sik2. Staurosporine, a non-selective Sik inhibitor, downregulated p-Sik2 level. However, NaCl displays no effect on total Sik2 expression during the 2-h period.

To determine whether SIRT3 is a target of Sik2, we next performed a co-immunoprecipitation assay. Endogenous Sik2 is co-immunoprecipitated with SIRT3 in brown adipocyte lysates (Fig. 7d). To examine the role of Sik2 in regulating BAT phenotype conversion, brown adipocytes were infected with lentivirus encoding short hairpin (sh) RNA against Sik2 (Sik2-shRNA), which reduced Sik2 protein expression by 80%. Notably, Sik2 knockdown decreased SIRT3 level, the effect that was exacerbated by HS (Fig. 7e, f). Sik2 depletion also increased K83 acetylation of PDHA1 (Fig. 7g, i), accompanied with depressed PDH activity (Fig. 7h). The oxygen consumption rate was significantly inhibited in Sik2-shRNA infected cells (Fig. 7j, k). Similar trends were observed in the levels of the mitochondrial proteins PGC-1α, UCP-1, complex I, the lipid droplet-binding proteins perilipin-1 and Cidea relative to cells transduced with a control scrambled shRNA (Fig. 7l–o). These results indicate that Sik2 signalling is involved in SIRT3-induced mitochondrial biogenesis.

### SIRT3 deficiency alters lipid metabolite levels in response to HS

**Fig. 8:**
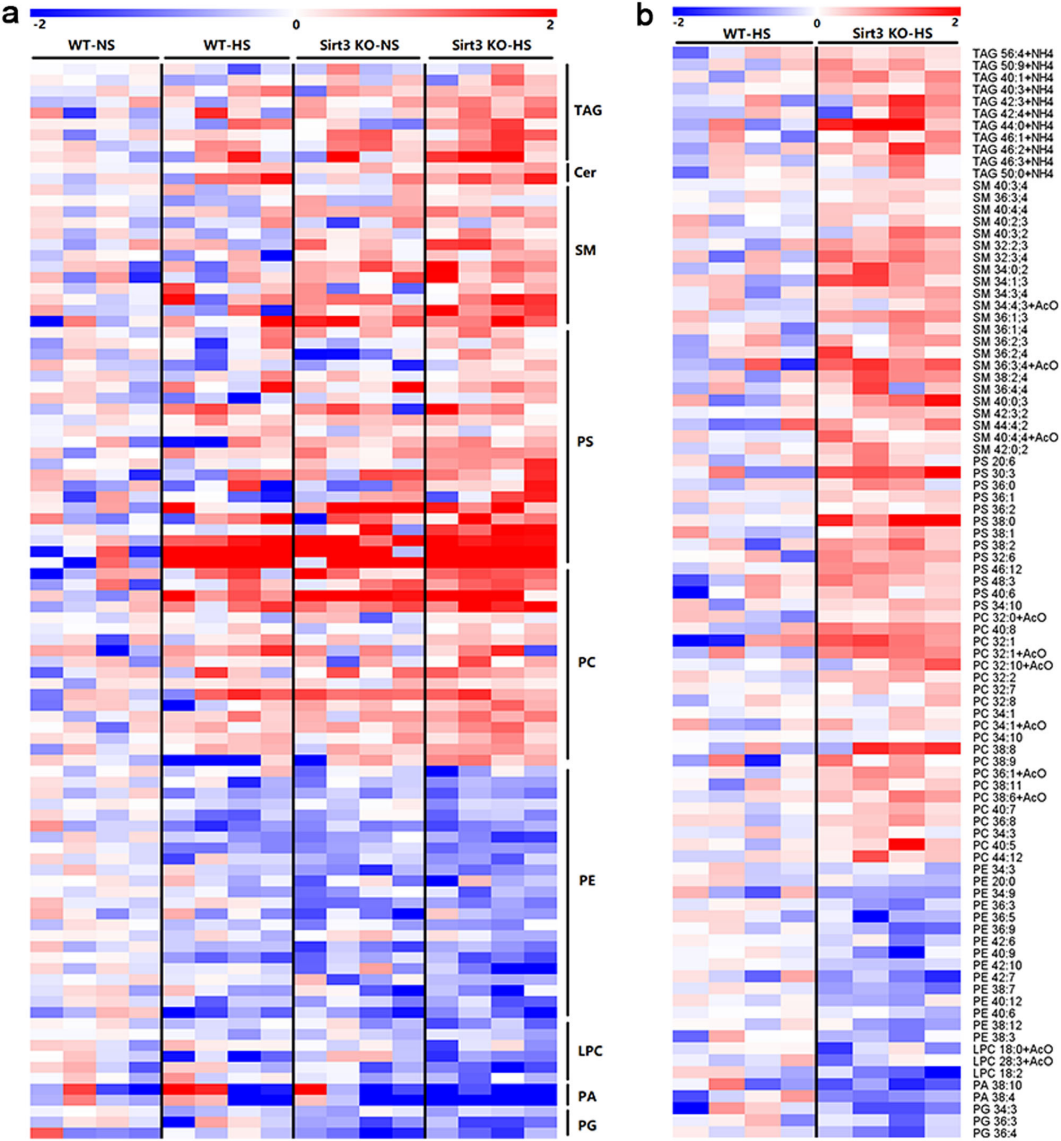
SIRT3 deficiency affects lipidome profile of BAT under HS conditions. **a** Lipids were extracted from BAT in WT and SIRT3-KO mice with or without HS intake. Heatmap visualization of TAG, Cer, SM, PS, PC, PE, LPC, PA and PG were determined via LC–MS. Data in the heatmap is *z*-scored. **b** Lipids were extracted from BAT in WT and SIRT3-KO mice with HS intake. Data in the heatmap is *z*-scored and sorted at the subclass level based on the total hydrocarbon chain length and then the total of double bonds in hydrocarbon chains

To determine the effects of HS intake on the lipidome profile of BAT, we analysed the lipid content and composition of BAT by liquid chromatography–mass spectrometry (LC–MS). A lipidome analysis using BAT samples from WT and SIRT3-KO mice under NS or HS revealed 309 lipid species from 9 (sub)-classes including triacylglycerol (TAG), ceramide (Cer), sphingomyelin (SM), phosphatidylserine (PS), phosphatidylcholine (PC), phosphatidylethanolamine (PE), Lysophosphatidylcholine (LPC), phosphatidic acid (PA) and phosphoglycerate (PG). As illustrated by the heatmap (Fig. 8a, b), the total TAGs were significantly increased in BAT from SIRT3-KO mice as compared with WT mice consistent with the results of the increased energy storage during BAT conversion to WAT (Fig. 8). HS led to TAG species with chain lengths of 40–56 carbons increased in SIRT3-KO mice than in WT mice. Similarly, higher levels of Cer, SM, PS and PC were detected in BAT from SIRT3-KO as compared with WT mice. Further analysis showed that this increase is caused by increased levels of SM containing 32–42 carbons, PS containing 20–48 carbons and PC containing 32–44 carbons in BAT from SIRT3-KO as compared with WT mice. In contrast, the levels of PE, LPC, PS and PC were significantly decreased in BAT from SIRT3-KO as compared with WT mice. HS decreased PE containing 20–42 carbons, LPC containing 18–28 carbons, PA containing 38 carbons, PG containing 36 carbons in SIRT3-KO mice more than in WT mice. These data indicate that SIRT3 regulates structural lipid content and composition in BAT.

## Discussion

The results of this study demonstrate that (1) SIRT3 level was downregulated in BAT under HS conditions. (2) Loss of SIRT3 exacerbated HS-induced BAT phenotype conversion. (3) SIRT3 interacted with and deacetylated PDHA1 lysine 83. (4) Targeting Sik2/SIRT3 axis may improve HS-induced BAT remodelling.

Consistent with the role of SIRT3 as a determinant of the BAT phenotype^19^, we observed a progressive loss of BAT morphological characteristics, which was accompanied by downregulation of SIRT3 under HS conditions. This resulted in a WAT-like morphology, with larger lipid droplets and fewer mitochondria. Interestingly, SIRT3-KO mice showed increased water consumption during HS intake, while blood glucose level and food intake were comparable with WT mice. This suggests that higher salt intake is positively associated with BAT remodelling independent of blood glucose and energy intake, as previously reported^27^. We speculate that BAT morphological abnormalities and thermogenic dysregulation contribute to the impairment of SIRT3 expression under HS conditions.

An abundance of mitochondria and multilocular lipid droplets are responsible for the characteristic appearance of BAT. In the current study, we showed that loss of SIRT3 is accompanied by suppression of mitochondrial biogenesis, as evidenced by decreases in mitochondrial DNA copy number and expression of mitochondrial proteins and the transcription factor PGC-1α, which is a major regulator of brown adipocyte function and may play an important role in activating BAT thermogenesis^28^. We and other researchers have shown that increased PGC-1α induces the transcription of Nrf1 and Nrf2, leading to upregulation of TFAM, which translocates into mitochondria and stimulates mitochondrial DNA replication and gene expression^29^. In vitro studies have found that SIRT3-deficient brown adipocytes had reduced UCP-1 expression and mitochondrial biogenesis, while a HS diet-induced decrease in SIRT3 impaired mitochondrial biogenesis in BAT, which was correlated with obesity. Restoring SIRT3 level partly reversed the mitochondrial biogenesis phenotype under HS conditions, consistent with previous findings that SIRT3 promoted mitochondrial biogenesis and activated UCP-1^30^ in response to cold exposure and caloric restriction^31^.

Brown adipocytes are morphologically distinct from white adipocytes except that they have more mitochondria and store lipids as multilocular droplets. In this study, we found that HS intake caused an increase in lipid droplet size in brown adipocyte, which was associated with reduced perilipin-1 and Cidea levels, especially in SIRT3-KO mice. Perilipin-1 exclusively localises at the surface of lipid droplets, which may promote lipid droplet formation^32^. Our previous study showed that loss of perilipin-1 increased lipid droplet size^7^, while others have reported that overexpression of perilipin-1 in white adipocytes reduced the size, resulting in a BAT-like phenotype^33^. Thus, perilipin-1 functions as a critical regulator of lipid droplet structure. Cidea, a member of cell death-inducing DNA fragmentation factor-alpha-like effector (CIDE) family, is an important regulator of lipid storage in brown adipocytes^34^. Perilipin-1 cross talk with lipid CIDE family proteins promotes droplet formation in adipocytes^35^. Our data showed that HS inhibited Cidea expression; however, SIRT3 had no effect on Cidea acetylation (data not shown). A recent study reported that acetylation of Cidec is vital for lipid storage in WAT^36^; Cidec is highly expressed in mature WAT; it was almost undetectable in BAT. It is worth clarifying the interaction between SIRT3 and Cidec in a future study.

PDHA1 is the first component enzyme of the pyruvate dehydrogenase (PDH) complex that transforms pyruvate, via pyruvate decarboxylation, into acetyl-CoA that is subsequently used by both the citric acid cycle and oxidative phosphorylation to generate ATP. In the present study, to identify the role of SIRT3 in the regulation of PDHA1, we performed co-immunoprecipitation using an antibody directed against endogenous SIRT3 and PDHA1. In addition, gain- or loss-of-function of SIRT3 altered the acetylation status of PDHA1, and acetylation/deacetylation of PDHA1 changed PDH activity, which was correlated with a change in UCP-1 and PGC-1α level. This suggests that PDHA1 is a potential substrate for SIRT3. The result seems reasonable since it has been proposed that the acetylome plays a central role in fine-tuning of downstream deacetylation targets. Mass spectrometry-based analysis revealed that PDHA1 is acetylated on multiple lysine residues. Among recent studies, Ozden et al. reported that SIRT3 deacetylates PDHA1 lysine 321 (K321) and increases in PDH activity^16^. Jing et al. indicated that lysine 336 (K336) of PDHA1 is a substrate of SIRT3, which can alter the phosphorylation status of PDH^37^. Our results suggest that PDH lysine 83 (K83) determined PDH activity, loss of SIRT3 and hyper acetylation of PDHA1 K83 resulted in mitochondrial dysfunction as well as aberrant thermogenesis in BAT under HS. It is speculated that PDH activity was regulated by different reversible acetyl-lysines responding to different environmental nutrient conditions. In addition, DCA is an inhibitor of PDK, and DCA treatment partially restored PDH activity, increased oxygen consumption rate and expression of the thermogenic markers in SIRT3-null brown adipocytes under HS. It is indicated that SIRT3 determines brown adipocytes phenotype via mediating pyruvate dehydrogenase activity.

SIK belongs to the AMPK-related kinases and shares similar substrates as AMPK. Sik2 is highly enriched in interscapular BAT. Phosphorylation of SIK2 at Thr175/Thr163 is the hallmark of its kinase activation^38,39^. However, the role of HS in lipid metabolism and fat deposition is debated. Sik2 may function similarly to AMPK for turning off lipogenesis. However, others have demonstrated that Sik2 KO mice exhibited enhanced adaptive thermogenesis respond to β-adrenergic stimuli^40^. In the current study, Sik2 activity decreased under HS stress. Furthermore, HS inhibited AICAR-induced Sik2 activation. Endogenous Sik2 and SIRT3 were found to directly interact, and depletion of endogenous Sik2 reduced SIRT3 expression in brown adipocytes. Conversely, loss of SIRT3 had no effect on Sik2 expression. The inhibitory effect of Sik2 on PGC-1α and perilipin-1 levels likely occurs through Sik2, which is presumed to be a downstream target of SIRT3. In addition, we also found that HS caused complete AMPK suppression but only partially reduced SIRT3 level. Thus, HS intake may induce BAT remodelling via downregulation of Sik2/ Sirt3 signalling.

We identified changes in the BAT lipidome that were associated with HS-induced BAT remodelling and were especially pronounced in the absence of SIRT3. It was previously reported that obesity-induced increases in BAT TAG levels were associated with a reduction in BAT mitochondrial thermogenesis. On the other hand, exercise decreased TAG content in BAT by stimulating this process, demonstrating that BAT has a high capacity for glucose disposal and TAG clearance^41,42^. We propose that HS dysregulates mitochondrial uncoupling, resulting in limited TAG utilisation and TAG accumulation in BAT. We also confirmed that SIRT3 plays an important role in adaptive thermogenesis in BAT, and that SIRT3-mediated deacetylation and activation of PDHA1 is an important cellular response for increasing mitochondrial metabolism. Thus, HS-induced SIRT3 inhibition leads to mitochondrial dysfunction in BAT.

Glycerophospholipids are the major components of plasma and mitochondrial membranes. Previous work has shown that changes in glycerophospholipids can affect membrane structure and/or signalling pathways. In the current study, we observed significant remodelling of glycerophospholipids in response to HS. Our lipidomics analysis revealed that the abundance of PE was reduced whereas that of PC was increased under HS conditions, possibly due to HS-induced suppression of SIRT3. Sphingolipids act as lipid organisers of the plasma membrane^43,44^. Abnormalities in sphingolipid metabolism have been linked to the pathogenesis of obesity/diabetes and atherosclerosis^45,46^. HS activated sphingolipid metabolism in BAT, which contributes to adipose hyperplasia^47^. Similarly, SIRT3 ablation exacerbated the dysregulation of sphingolipid metabolism. These results indicate that the selective remodelling of glycerophospholipid and sphingolipid subspecies have functional implications during HS stress. Future studies should focus on examining the contributions of specific phospholipid subspecies to the modulation of the thermogenic response in BAT.

In summary, this study suggests that the SIRT3 deficiency exacerbates the impairment of BAT mitochondrial biogenesis and lipid droplets size, and that targeting Sik2/SIRT3/PDHA1 axis may improve high-salt-induced BAT remodelling (Supplementary Fig 3). These findings provide a basis for future studies on lipid metabolism pathways that are altered by consuming a HS diet and how these contribute to disease states.

## Supplementary information

Supplemental Material 1

Supplementary figure 2

supplementary fig 3

Supplementary figure legends
