# Supplementary figures and images for: Sirtuin 3-mediated pyruvate dehydrogenase activity determines brown adipocytes phenotype under high-salt conditions
Source: Cell Death Dis. 2019 Aug 14;10(8):614. doi: 10.1038/s41419-019-1834-4 (PMC6692335; doi:10.1038/s41419-019-1834-4)

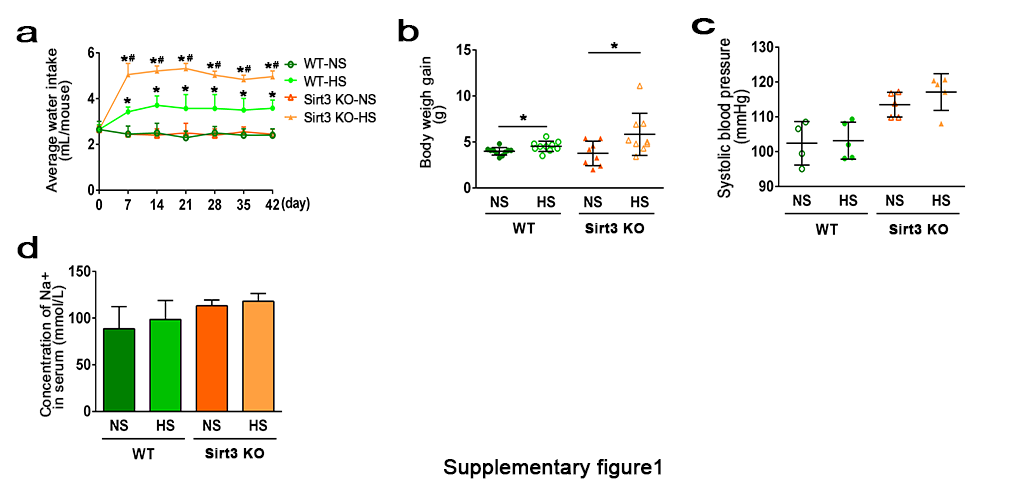

Supplement: Supplementary file 1 — Supplemental Material 1 [file 41419_2019_1834_MOESM1_ESM.tif]

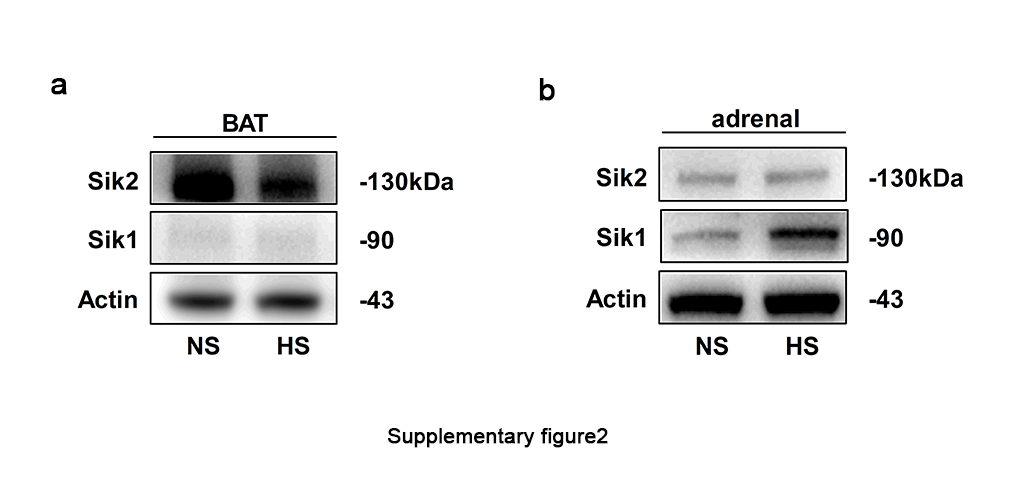

Supplement: Supplementary file 2 — Supplementary figure 2 [file 41419_2019_1834_MOESM2_ESM.tif]

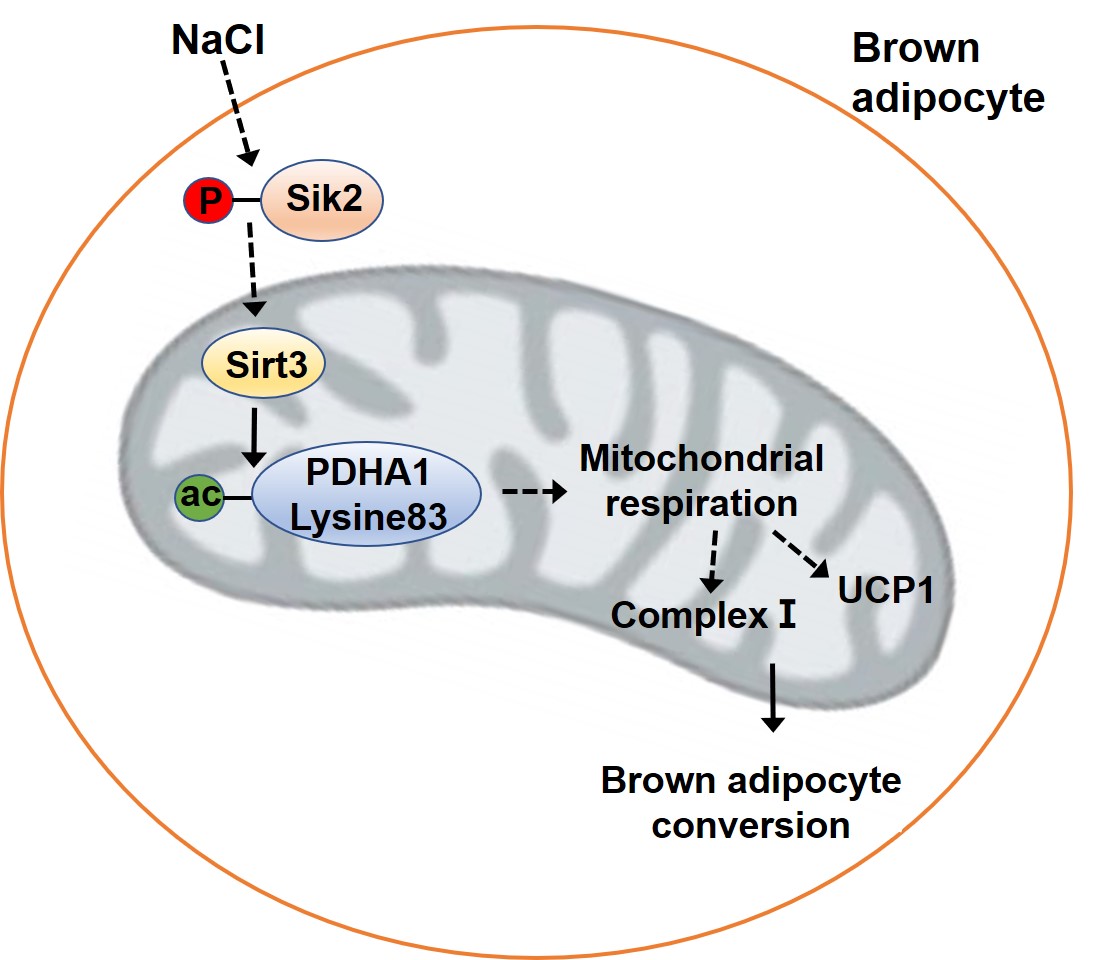

Supplement: Supplementary file 3 — supplementary fig 3 [file 41419_2019_1834_MOESM3_ESM.jpg]
