# Supplementary material for: Sirtuin 3-mediated pyruvate dehydrogenase activity determines brown adipocytes phenotype under high-salt conditions
Source: Cell Death Dis. 2019 Aug 14;10(8):614. doi: 10.1038/s41419-019-1834-4 (PMC6692335; doi:10.1038/s41419-019-1834-4)
Supplement: Supplementary file 4 — Supplementary figure legends [file 41419_2019_1834_MOESM4_ESM.docx]

**Supplementary Figure 1.** Physiological characteristics of WT and Sirt3-KO mice under HS conditions. (a) Daily water consumption by WT and Sirt3-KO mice during HS intake (n = 10/group). (b) Body weight gain, (c) systolic blood pressure and (d) serum sodium level of WT and Sirt3-KO mice (n = 10/group). Data represent mean ± SEM; **p*< 0.05 vs. genotype-matched NS mice. **^#^***p*< 0.05 vs. WT HS mice.

**Supplementary Figure 2.** Sik1 and Sik2 protein levels in BAT and adrenal gland under HS conditions. (a) Representative western blots of Sik1and Sik2 in BAT. (b) Representative western blots of Sik1 and Sik2 in adrenal gland.

**Supplementary Figure 3.** SIRT3 deficiency exacerbates the impairment of BAT mitochondrial biogenesis and lipid droplets size, and that targeting Sik2/Sirt3/PDHA1 axis may improve high-salt-induced BAT remodeling.
